# A rare association of Vagus Nerve Schwannoma and Pheochromocytoma: A case report

**DOI:** 10.1016/j.amsu.2022.103475

**Published:** 2022-03-04

**Authors:** Marouane Harhar, Abdelhakim Harouachi, Tariq Bouhout, Badr Serji, Tijani EL Harroudi

**Affiliations:** a Surgical Oncology Department, Regional Oncology Center, Mohammed VI University Hospital, Oujda, Morocco; b Mohammed First University Oujda, Faculty of Medicine and Pharmacy Oujda, Oujda, Morocco

**Keywords:** Vagus nerve, Schwannoma, Pheochromocytoma, Resection, Immunohistichimical staining

## Abstract

Vagus nerve schwannoma is a very rare benign nerve tumor. Pheochromocytoma is a rare, mostly benign tumor of the adrenal medulla with a large clinical spectrum. Their association is uncommon. The management of both tumors depends solely on surgery. The surgery of vagal schwannomas is particularly challenging considering the anatomical compositions of the area. Here, we report a case of a 76 year-old patient with cervical vagal schwannoma and benign pheochromocytoma association. We discuss the diagnosis and the surgical management of these tumors.

## Highlights

• Vagus nerve schwannoma is a very rare benign nerve tumor.
• Pheochromocytoma is a rare catecholamine-secreting tumor that originates in the adrenal medulla and is responsible for secondary hypertension.
• We describe the case of a patient with pheochromocytoma, in whom a cervical vagal schwannoma was discovered incidentally.

## 1 Introduction

Cervical schwannoma of the vagus nerve is an extremely rare neurogenic benign tumor with slow-growing evolution. It is majorly asymptomatic and rarely undergoes malignant transformation [1]. The pre-operative diagnosis is difficult and its surgical management is even harder due to the proximity of vascular structures and risk of postoperative complications such as hoarseness of the voice [2].

Pheochromocytoma is a rare catecholamine-secreting tumor that originates in the adrenal medulla and is responsible for secondary hypertension [3]. Its diagnosis is suspected clinically and confirmed by increased serum or urinary catecholamines. Radiological imaging helps locate the tumor and surgery remains the key of treatment after medical preparation.

To our best knowledge, few cases of their association have been reported in the literature. Here, we describe the case of a patient with pheochromocytoma, in whom a cervical vagal schwannoma was discovered incidentally. Based on a literature review, we discuss the diagnosis, pathogenesis, surgical management and genetic disorders of this association. This case report follows SCARE guidelines 2020 [13].

## 2 Presentation of case

We report a case of a 76 year-old man, with a history of Type 2 diabetes on oral antidiabetic drugs. The patient was presented in the emergency department with the classic triad of: headache, sweating, and heart palpitation and hypertension. He was non-smoker and has no other medical or family history. Laboratory tests were done, including 24-h urine fractioned metanephrine and catecholamine, were elevated. Twenty days earlier, he had been hospitalized to the regional hospital for a hypertensive crisis at 240/105 mmHg associated with headaches, palpitations and hot flashes. On physical examination, he had normal cardiopulmonary and neurological systems. Notably absent were symptoms of nausea, vomiting, bloating, and change in bowel habit or weight loss.

**Fig. 1:**
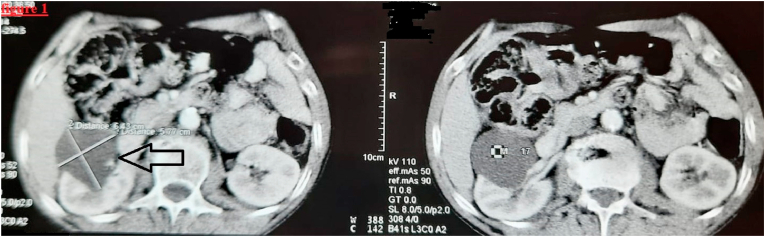
abdominal computer topography scan shows a heterogeneous singe mass of the right adrenal gland, measuring 64 × 57 mm (arrow).

**Fig. 2:**
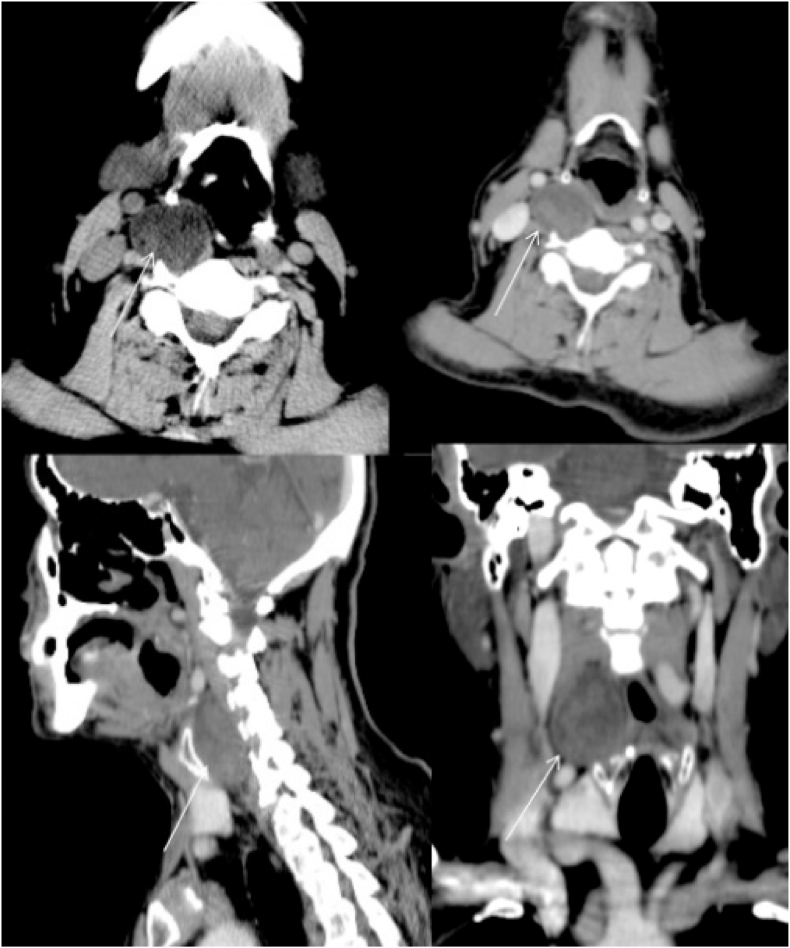
Acervical computer topography demonstrates a right well-defined retro laryngeal and pre-vertebral mass, measuring 30 × 30 mm (arrow).

Cervical and thoraco-abdominal computed tomography (CT scan) revealed a large single mass of the right adrenal gland. It was heterogeneous with areas of necrosis and cystic change, measuring 64 × 57 mm (Fig. 1). Besides, a cervical lesion was found incidentally. This lesion was a right well-defined retro laryngeal and pre-vertebral mass, measuring 30 × 30 mm, with low contrast enhancement and external displacement of the carotid artery and jugular vein (Fig. 2). No metastases were found. The level of serum tumor marker, were within the normal range.

Based on the clinical and radiological findings, the therapeutic approach in this case consisted of an urgent open right adrenalectomy. The patient was ready to undergo surgery by an experienced surgeon after medical preparation with α-adrenergics and hypertension control. The exploration of the right adrenal gland confirmed the presence of a 9 cm cystic mass that was carefully extracted. No extra-adrenal nodules were seen. The procedure lasted 90 min, with an intra-operative diuresis of 600 ml and bleeding loss estimated at 100 cc. The post-operative recovery of the patient was without incident, and two weeks later, we noted the normalization of blood pressure and urinary catecholamines.

**Fig. 3:**
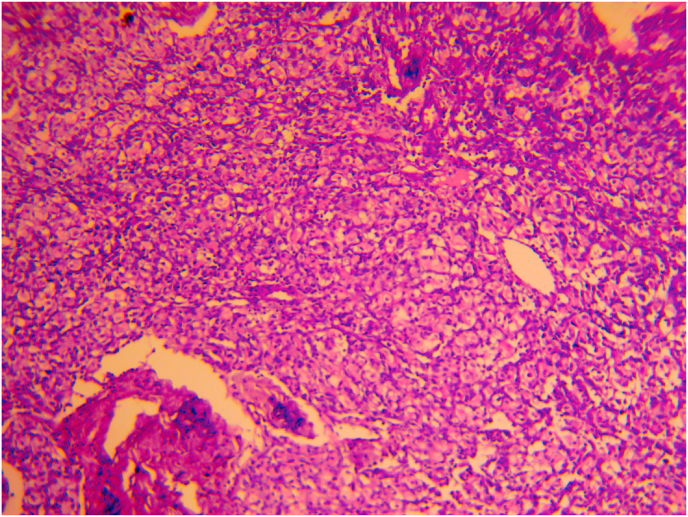
Microphotograph showing a benign pheochromocytoma of the adrenal gland.

The histopathological examination confirmed the diagnosis of pheochromocytoma, (Fig. 3).

The patient refused the exploration of his cervical lesion associated with pheochromocytoma and was lost to follow-up. Six months later, the patient presented herself in our department with complaint of headaches and cervical swelling. Vital parameters and all laboratory investigations, including blood exam, kidney function and urinalysis, were within normal limits.

**Fig. 4:**
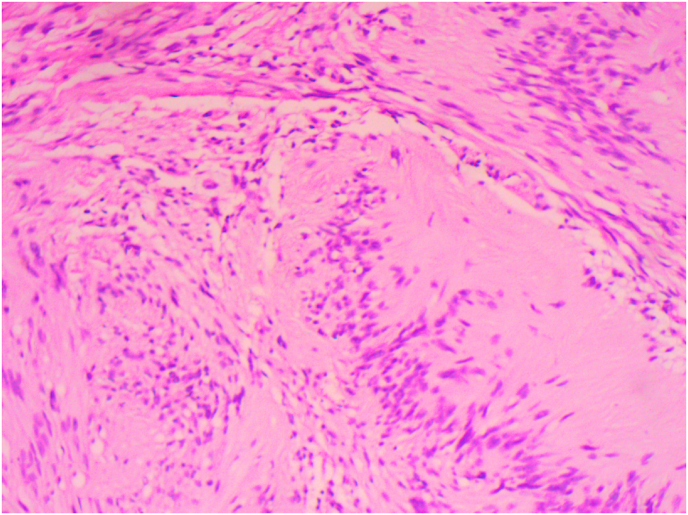
Microphotograph showing a cervical shwannoma arising from the vagus nerve.

Under general anesthesia, the patient underwent a cervicotomy with an excision of the cervical mass, with uneventful postoperative course. The histopathological examination confirmed the diagnosis of schwannoma arising from the vagus nerve (Fig. 4).

The patient currently continues to be asymptomatic and disease-free 24 months later. There was no evidence of recurrence.

Taking the cunt association of the pheochromocytoma and the cervical schwannoma, we suspected a possible genetic alteration. A genomic analysis was indicated, but the patient was refused this genetic testing.

## 3 Discussion

Cervical vagal schwannomas are extremely rare slow-growing tumors originating from the vagus nerve. They are covered with Schwann cell sheath [5]. Schwannomas constitute 25–45% of tumors of the head and neck [6]. Most schwannomas of the vagus nerve are benign tumors. They affect genders equally between the third and sixth decades of life [4].

These tumors are usually asymptomatic and present as a neck mass. If there are symptoms, they are majorly represented by hoarseness and occasionally a paroxysmal cough when palpating the mass, which is a pathognomonic sign of vagal schwannoma [4].

Cervical computed tomography (CT) with contrast can help in the diagnosis of this tumor, but magnetic resonance imaging (MRI) remains the tool of choice for the diagnosis and determination of the nerve origin, but also for providing pre-operative information useful in planning surgical treatment [7]. Fine-needle aspiration cytology (FNAC) has low specificity [4].

The gold standard of treatment is the surgery that consists of intracapsular enucleating of the tumor in order to minimize nerve damage, guided by a nerve stimulator and the surgical microscope. Conventional extracapsular excision with end to end anastomosis has been done with mixed results, because it can damage the normal fascicles during the dissection of the capsule [8,9]. The main post-operative complication is vocal cord palsy.

Histologically, schwannoma typically show two main patterns, a very cellular area made of spindle cells with palisading of cell nuclei (Antoni A) and a less cellular area (Antoni B) with strong staining of S-100 protein.

Given the benign nature of most vagal schwannomas and their slow-growing evolution, a conservative approach should always be considered. In our case, the patient started to show cervical pain and hoarseness leading to the decision of surgical excision.

Pheochromocytoma is a rare neuroendocrine tumor originating in the adrenal gland medulla and producing catecholamines and other neuropeptides that are responsible for secondary hypertension. They are often seen between the 3rd and 5th decades of life. According to literature, 10–25% of the cases of pheochromocytoma can be associated with genetic and familial syndromes (multiple endocrine neoplasia type 2 (MEN 2), type 1 neurofibromatosis and Von-Hippel-Landau disease in younger ages [10].

The typical triad of headache, sweating and heart palpitations guides the diagnosis and elevated plasmatic or urinary metanephrine and normetanephrine confirms it. Computed tomography (CT) and magnetic resonance imaging (MRI) help locate the tumor [11].

Surgical excision remains the best treatment option after a thorough medical preparation with α adrenergics and eventually β adrenergics.

The association of both tumors in a patient is very rare, and to our best knowledge, only few cases have been reported [12].

Given the presence of pheochromocytoma, the surgery should focus first on the adrenal gland. The cervical vagus schwannoma can be postponed. A testing for genetic disorders should be suggested to our patient. The major limitations in term of diagnosis of this association are the determination of the nature of these tumors, and the surgical management.

## 4 Conclusion

This case report sheds light on the rarity of cervical vagus schwannoma and its association with benign pheochromocytoma. A thorough study of this association should be done, including researching for genetic disorders in order to comprehend this association.

## Ethical approval

No ethical approval necessary.

## Sources of funding

All sources of funding should be declared as an acknowledgement at the end of the text. Authors should declare the role of study sponsors, if any, in the collection, analysis and interpretation of data; in the writing of the manuscript; and in the decision to submit the manuscript for publication. If the study sponsors had no such involvement, the authors should so state.

The author(s) received no financial support for the research, authorship and/or publication of this article.

## Author contribution

**Dr Harhar Marouane:** Have written the article, have consulted the patient, prescribed all of the tests and prepared the patient for surgery and participated in the surgery. **Dr Harouachi Abdelhakim:** have helped writing the article, data collection. **Pr Bouhout Tariq** (oncology surgery professor)**:** have supervised the writing of manuscript. **Pr Serji Badr** (oncology surgery professor): have supervised the writing of the paper. **Pr El Harroudi Tijani** (oncology surgery professor): Writing, review and editing of the manuscript, and had been the leader surgeon of the case.

## Consent

Written informed consent was obtained from the patient for publication of this case report and accompanying images. A copy of the written consent is available for review by the Editor-in-Chief of this journal on request.

## Trial registry number

Our paper is a case report; no registration was done for it.

## Guarantor

Harhar Marouane.

## Provenance and peer-review

Not commissioned, externally peer reviewed.

## Declaration of competing interest

The authors declared no potential conflicts of interests with respect to research, authorship and/or publication of the article.
